# Monitoring and regulation of learning in medical education: the need for predictive cues

**DOI:** 10.1111/medu.13267

**Published:** 2017-03-23

**Authors:** Anique B H de Bruin, John Dunlosky, Rodrigo B Cavalcanti

**Affiliations:** ^1^ Maastricht UniversityMaastrichtThe Netherlands; ^2^ Kent State UniversityKentOhioUSA; ^3^ University of TorontoTorontoOntarioCanada

## Abstract

**Context:**

Being able to accurately monitor learning activities is a key element in self‐regulated learning in all settings, including medical schools. Yet students’ ability to monitor their progress is often limited, leading to inefficient use of study time. Interventions that improve the accuracy of students’ monitoring can optimise self‐regulated learning, leading to higher achievement. This paper reviews findings from cognitive psychology and explores potential applications in medical education, as well as areas for future research.

**Cognitive Psychology:**

Effective monitoring depends on students’ ability to generate information (‘cues’) that accurately reflects their knowledge and skills. The ability of these ‘cues’ to predict achievement is referred to as ‘cue diagnosticity’. Interventions that improve the ability of students to elicit predictive cues typically fall into two categories: (i) self‐generation of cues and (ii) generation of cues that is delayed after self‐study. Providing feedback and support is useful when cues are predictive but may be too complex to be readily used.

**Application to Medical Education:**

Limited evidence exists about interventions to improve the accuracy of self‐monitoring among medical students or trainees. Developing interventions that foster use of predictive cues can enhance the accuracy of self‐monitoring, thereby improving self‐study and clinical reasoning. First, insight should be gained into the characteristics of predictive cues used by medical students and trainees. Next, predictive cue prompts should be designed and tested to improve monitoring and regulation of learning. Finally, the use of predictive cues should be explored in relation to teaching and learning clinical reasoning.

**Conclusions:**

Improving self‐regulated learning is important to help medical students and trainees efficiently acquire knowledge and skills necessary for clinical practice. Interventions that help students generate and use predictive cues hold the promise of improved self‐regulated learning and achievement. This framework is applicable to learning in several areas, including the development of clinical reasoning.

## Introduction

Monitoring decisions and behaviour is a central part of medical professionals’ daily practice. Whether to ask for advice from a senior colleague, prescribe a particular medication or discharge a patient home, most clinical management choices involve physicians self‐monitoring. Self‐monitoring also contributes to performance in medical *education*, where medical students must learn and be able to adaptively use massive amounts of information. As students work toward mastery, they rely on monitoring judgements of their knowledge in order to regulate learning, moving from concepts they judged that they know or have learned to focus on concepts they judged that they have not yet mastered. Table 1 provides an overview and explanation of the concepts that are central to this manuscript, with a specific focus on monitoring and regulation of learning and performance. To limit the scope, we focus specifically on the learning task or micro level, and refrain from discussing macro level issues related to students’ confidence in their general ability to self‐assess or self‐regulate (i.e. self‐efficacy).

**Table 1:** An overview of concepts, their explanation and standard measures critical to research on monitoring and regulation of learning

Concept	Definition	Standard measures
Monitoring of learning	Evaluating how well one has learned or understood certain information (e.g. a textbook chapter)	Monitoring judgements (e.g. judgements of learning or understanding, either prior to, during or after learning)
Monitoring of performance	Evaluating how well one has performed a certain task (e.g. taking an examination, diagnosing a patient or performing a lumbar puncture)	Monitoring judgements (e.g. predictions or post‐dictions of performance)
Regulation of learning	Evaluating what next steps need to be taken to achieve learning goals	Regulation decisions (e.g. deciding which paragraphs or chapters need restudying)
Regulation of performance	Evaluating what next steps need to be taken to achieve performance goals	Regulation decisions (e.g. deciding which sub‐skills need to be practised further)

All these judgements are self‐judgements. Regulation judgements are typically based on monitoring judgements: a student bases her regulation decisions on how she monitors her learning or performance.

The *accuracy* of monitoring judgements is fundamental to both medical education and medical practice. Inaccurate judgements are known to undermine achievement.^1^ Multiple studies have also reported that the relation between monitoring judgements and performance among practising physicians is mediocre at best.^2^, ^3^, ^4^, ^5^ Davis *et al*.^6^ provide a review of 20 empirical studies comparing physicians’ self‐assessments of performance with external observations and identify 13 in which there was little, no or an inverse relation between these variables. We argue that improving the accuracy of monitoring judgements by medical professionals should start at the beginning of training, ideally by helping medical students develop more accurate monitoring of their learning. Monitoring judgements are known to be inferential in nature and are based on cues such as perceived difficulty of the task or interest in the topic.^7^ Improving monitoring judgements therefore entails improving the quality of the cues that students use when making judgements of learning.

In this cross‐cutting edge paper, we first describe cognitive and educational psychology studies that show how learners can improve the accuracy of their monitoring judgements.^8^, ^9^ We explore the literature regarding cues that learners use in judging their learning (termed ‘cue utilisation’) and the predictive accuracy of these cues in informing judgements of learning (termed ‘cue diagnosticity’). We then propose possible translations of this work to medical education, specifically to acquiring conceptual knowledge and to developing clinical reasoning skills. Throughout the manuscript, we refer to learning as the cognitive act of processing and acquiring knowledge and skills, and to performance as demonstrating knowledge and skills on a certain task (e.g. taking a test or performing a lumbar puncture). The question we address is: How does research on monitoring and regulation of learning and monitoring of performance inform new directions in medical education?

## Monitoring and Control: A Metacognitive Framework

**Figure 1:**
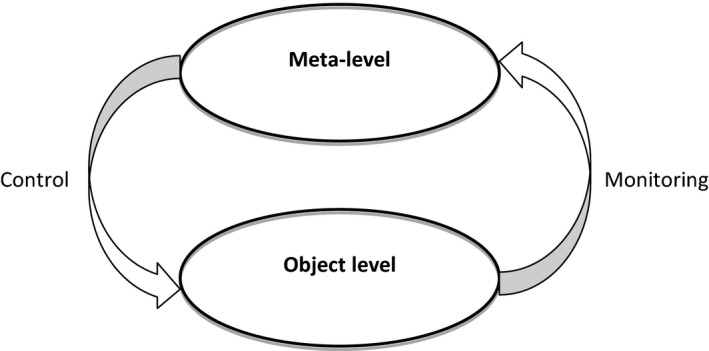
The intricate relation between monitoring and control (Nelson & Narens)^9^. Reprinted by kind permission of Elsevier.

Judging how well a topic has been mastered represents a form of metacognitive monitoring. *Monitoring* here pertains to assessing one's knowledge, and for medical students, such monitoring may involve judging one's confidence in how well one understands a topic (e.g. physiology of the cardiovascular system), how well one can accurately diagnose a clinical case or how well one can perform a procedure (e.g. a lumbar puncture). As emphasised by Nelson,^9^ monitoring is used to *control* or *regulate* learning and performance by, for example, steering learning efforts to areas in need of further development (see Fig. 1 for a visual representation).

Monitoring and control have reciprocal influences: monitoring informs a student about whether (and how) to control their learning and performance, and further progress toward a learning goal can be used to update monitoring. Thus, while studying a textbook chapter (the object level, Fig. 1), a student may not be confident about her understanding of concepts in the chapter (the meta‐level), and hence decide to use more time studying specific text segments (control) in the hope of boosting her understanding. Such a monitoring‐control feedback system is evident in all general frameworks of metacognition and highlights the vital role of monitoring accuracy as the basis of effective regulation and performance.

The quality of monitoring judgements is usually termed ‘monitoring accuracy’ and is determined by establishing the relation between monitoring judgements and actual learning or performance. To better understand how monitoring can impact regulation and performance, it is vital to distinguish between two kinds of monitoring accuracy: absolute accuracy and relative accuracy (for details and further references, see Chapter 3, Dunlosky & Metcalfe).^10^

*Absolute accuracy* refers to the degree to which the magnitude of one's monitoring differs from actual performance. For example, if a student expects to obtain 80% in an upcoming examination about anatomy, the student would be viewed as having perfect absolute accuracy if he or she scored 80% in the examination. If the examination performance was below 80%, the student would be considered overconfident, and if performance was above 80%, the student would be considered underconfident. Absolute accuracy can be measured per learning task (e.g. studying a textbook chapter on blood circulation) or across a set of learning tasks or items (e.g. when taking a course examination). Overconfidence in learning can lead students to prematurely stop studying and perform poorly on subsequent tests,^1^ and overconfident decision makers will rob themselves of the opportunity to revisit and correct poor decisions.

*Relative accuracy* refers to the degree to which students can discriminate between the differential learning for some materials versus others. In the anatomy example above, the student may judge this learning to be better for the ligaments of the knee than for the bones of the foot, as one example. If this differentiation matches actual performance on the examination, relative accuracy is high. Relative accuracy is usually determined through a correlation between monitoring judgements and actual performance. Excellent relative accuracy can also lead to effective regulation; for instance, when preparing for an examination, students with higher relative accuracy make better decisions about which materials to continue studying and hence perform better than students with poor relative accuracy, who may direct their study efforts in a less efficient fashion.^11^ Importantly, the two kinds of accuracy are not necessarily related: a student can show perfect relative accuracy and at the same time be overconfident.

## Monitoring Accuracy: Cue Diagnosticity and Cue Utilisation

With this in mind, the questions then arise: How do students monitor their learning and performance? How can monitoring accuracy be improved to foster effective regulation and performance? We begin by ruling out one possible mechanism, called direct access, for how people monitor their learning. As demonstrated by Koriat,^8^, ^12^ people do not have direct access to the quality of their cognitive states. When medical students judge how they have learned a set of critical terms, they will not be able to directly inspect the representation of the terms in their memories to make this judgement. Whether judging the strength of a memory or the accuracy of a decision, people are just not capable of directly assessing the quality of their cognitive states.

Our understanding of cognitive self‐monitoring is to a great extent based on the cue‐utilisation framework.^8^ According to this framework, monitoring judgements are based on a variety of cues, such as perceived difficulty of the task or familiarity with the task. These cues come to mind when students judge their learning or performance, and students use these cues to estimate whether they are learning and performing well. To illustrate, imagine interns evaluating a case of acute shortness‐of‐breath. After generating a differential diagnosis, the students judge the likelihood of each potential diagnosis. Now, without having access to the correct diagnosis, they must rely on cues that may inform their judgement of whether a diagnosis is correct. In this case, one cue may be the speed with which the diagnosis comes to mind, with faster decisions being associated with a higher likelihood of being correct. Yet another cue may be the prevalence of a particular disease, with less common diseases potentially leading to lower judged likelihood. Similarly, students’ familiarity with a particular condition (based on previous cases or teaching sessions) may also influence their judgement of whether they have the correct diagnosis. No doubt other cues are also available, and according to the framework, people will use one or more of these to form a monitoring judgement.

The accuracy of monitoring judgements then depends on the extent to which the available cues are predictive or ‘diagnostic’ of students’ actual learning or actual performance, a concept termed ‘*cue diagnosticity*’.^13^ The more accurate the cues used to gauge learning or performance, the more accurate the ensuing judgement. When a student uses familiarity with the condition as a cue, judgement accuracy depends on the extent to which familiarity is predictive of the student's ability to formulate the correct diagnosis. That is, if the student formulates better diagnoses for familiar compared with unfamiliar diseases, then familiarity is a predictive cue and should be used when monitoring performance. Note that the term ‘diagnostic cue’ is often used in the metacognitive literature to refer to cues that are ‘predictive’ of learning. However, this use does not relate in any way to the medical sense of the term, as in ‘suffering from a specific disease’. So as to prevent confusion, we have favoured the term ‘predictive cue’ throughout our discussions in this paper.

**Figure 2:**
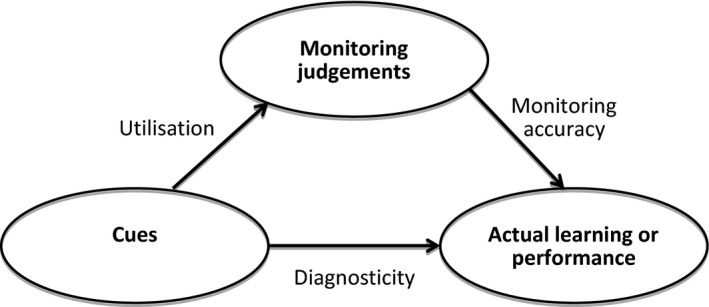
How monitoring judgements, cues and actual learning and performance relate and indicate monitoring accuracy, cue diagnosticity and cue utilisation

The shortness‐of‐breath example illustrates the second principle that drives accurate monitoring of learning and performance, which is *cue‐utilisation*. Typically, several cues are available, some of which are more predictive than others. Only when students select predictive cues and disregard non‐predictive cues will they arrive at an accurate monitoring judgement. In the example above, familiarity may be a more predictive cue than, say, prevalence of disease. If that is the case, it would be preferable for the student to base her judgement on familiarity. That is, accurate monitoring hinges upon students’ access to predictive cues and preferential *use* of predictive cues over other less informative cues when monitoring their learning or performance. This is illustrated in Fig. 2. *Cue diagnosticity* depends on the relation between cues and actual learning or performance, and as such informs students or trainees about their level of learning or performance. Accuracy of monitoring judgements measured by relating monitoring judgements to actual learning or performance then depends on whether students indeed used predictive cues.

## Improving use of Predictive Cues to Improve Monitoring and Regulation of Learning and Performance

Unfortunately, cues that arise when monitoring learning and performance are often not highly predictive of actual learning and performance. Reliance on non‐predictive cues leads to incorrect judgements, resulting in inaccurate monitoring. One pervasive, but often non‐predictive, cue is the speed with which people process information (also termed ‘fluency’), whether it involves the speed of learning new information or the speed of generating answers on a test. Such inferences based on processing speed are often misleading, because faster processing speed does not necessarily predict the quality of one's learning or performance, thus generating incorrect monitoring judgements.^14^

This example highlights inherent difficulties of accurately judging one's learning. Without direct access to learning outcomes, one resorts to using available cues to infer it, often inaccurately. To improve monitoring judgements, interventions are needed that aid students in identifying predictive cues (that is, improving *cue diagnosticity*) and that enhance the utilisation of these predictive cues. In the next section, we describe three types of interventions that can improve monitoring and aid in regulation of learning for medical students.

### Self‐generating predictive cues

Interventions to improve monitoring should target improving *availability* of predictive cues and *reliance on* these cues for monitoring learning and performance. These interventions can be considered ‘predictive cue prompts’ and are ideally inserted just prior to judging learning, to ensure maximum availability and reliable use. Possibly the most investigated predictive cue prompt is to have students engage in a task that simulates their learning task. Mental simulation then acts as a try‐out test that informs the student of his or her level of knowledge or performance. Simulations should activate the knowledge or skills the student possesses but need not be identical to the criterion task. Such simulations inform students about the quality of their knowledge or skills by providing them with predictive cues. One often‐cited example is having students generate keywords after studying a text.^11^, ^15^ In these keyword studies, students read a set of texts (usually around 1000 words long). After reading, students were instructed to formulate five keywords that captured the essence of each text. Generating keywords improved relative monitoring accuracy; the correlation between students’ monitoring judgements and their performance on a test of the texts was 0.71 (compared to only 0.25 without generating keywords). Presumably, keyword generation elicits students’ representation of what is called the ‘situation model of the text’,^16^ similar to what happens when writing a summary of a text.^11^ The situation model represents the student's knowledge of the text, including key concepts and their inter‐relations, integrated with the student's prior knowledge.^16^ Generating keywords is thought to act as a simulation of the situation model for each text, and helps students differentiate between well‐learned versus less well‐learned texts. Similar results have been found when generating summaries of texts,^17^ generating sentences when studying idioms^18^ and completing diagrams when studying causal relations.^19^

Research on the keyword effect has identified two crucial characteristics of effective predictive cue prompts: delaying judgements and self‐generation. Keyword generation only improves judgement accuracy if keywords are generated at a delay after study. The delay need not be longer than several minutes, or about as long as it takes for information to decay from short‐term memory. The necessity of this delay is explained by means of the monitoring‐dual‐memories hypothesis^20^: generating keywords *immediately* after studying a text uses information from short‐term memory. However, part of this information will decay from short‐term memory and not be available by the time of the test, and therefore immediate keyword generation is not an effective simulation because it would not yield predictive cues. Delayed keyword generation requires retrieval from long‐term memory, which more closely resembles memory available at the time of the test. This elicits more predictive cues and hence improves monitoring accuracy. The necessity of a delay has been repeatedly established in learning tasks based on declarative knowledge, but it is not clear whether this also applies to procedural or reasoning skills.

Another central aspect of predictive cue prompts is the need for self‐generation. Thiede *et al*.^21^ compared students who self‐generated keywords with students who read keywords generated by others, and found positive effects on judgement accuracy only in the first group. Apparently, the subjective experience of generating keywords is central to providing memory cues that are adequate predictors of text comprehension.

### Scaffolding self‐generative cue prompts

Circumstances can arise in which self‐generative cue prompts lead to predictive cues that are not interpretable by individual students. In work by Dunlosky, Hartwig, Rawson and Lipko^22^ students were asked to read science texts, each containing four key terms and their definitions that needed to be correctly learned.^22^ Sometime after studying, students were prompted to practice recalling the definitions. After recalling each definition, they judged how well they had performed. Puzzlingly, having students generate definitions of key terms prior to judging their performance still led to poor absolute monitoring accuracy – their judgements were substantially overconfident. This was likely to be because of students’ inability to self‐assess the quality of the definitions they had retrieved, instead relying on a less predictive cue, namely ‘accessibility’. That is, students were confident in their learning when they were able to access *any* definition, regardless of the quality of that definition. This difficulty in interpreting the outcome of self‐generative cue prompts indicates a shortcoming of these prompts.

When self‐generative cue prompts do not lead to *interpretable* predictive cues, one potential solution is providing external feedback or support. This should help students to evaluate the quality of the output of the cue prompt and thereby increase the availability of predictive cues. The kind of support required may not always be obvious and will require systematic research to identify. For instance, in the key term study, a first attempt to improve monitoring accuracy involved supplying students with the actual definition at the time of judging their self‐generated definitions. Doing so did reduce their overconfidence, but they still often said that they had correctly recalled a definition that was entirely incorrect. Overconfidence was nearly eradicated, however, when the support included the presentation of idea units in which each definition was separated into its smaller ideas. Students were asked to evaluate whether each idea unit was present in their self‐generated definition, and doing so dramatically reduced overconfidence. The support including idea units aided them in evaluating their output, which led to improved availability and use of the predictive cues the self‐generation task provided. What appears crucial is that the feedback or support relates to the *quality of generated content* and that it is presented in a way that enables students to self‐evaluate. In this example, the support using idea units acted like a standard rubric that highlighted the individual units required for excellent performance, so that students could evaluate their own performance with this easy‐to‐use rubric.

### When self‐generative cue prompts fail: explicit teaching of predictive cue use

For certain learning situations, it may be impossible to design an effective self‐generative cue prompt for a judgement task. We discuss two circumstances where this applies. First, when the predictive cues a learning task generates are experienced as counterintuitive, and second, in test‐taking situations. The first happens, for example, when the immediate cues students experience indicate a feeling of difficulty, although, in fact, the long‐term effects of the learning context are positive. Several learning strategies fall into this category, such as spacing learning sessions or self‐testing during learning. Spacing refers to distributing learning and repetition of information over several sessions (instead of cramming learning into one session), and self‐testing refers to taking practice tests on studied information (instead of merely restudying the original information).^23^, ^24^, ^25^, ^26^ The feeling of difficulty these learning strategies generates can lead students to judge their learning as low even though the strategies improve learning. Therefore, few students report using testing and spacing, despite robust evidence indicating their effectiveness in promoting learning.^23^ The so‐called ‘desirable difficulties’ these manipulations introduce are hardly ever recognised by students.^23^, ^24^, ^25^, ^26^ In such cases, students need to be directly taught about the positive effect of these strategies and the counterintuitive nature of the predictive cues they generate. Research by Pressley, Levin and Ghatala 27 demonstrated that persuading students to use effective learning strategies entails both learning of *and* practice with the strategies.

Second, the literature reviewed above on effective interventions focused on monitoring *during* learning. However, students must be able to accurately monitor *performance* too (e.g. when making clinical decisions or taking a test). Consider a test‐taking situation: How do students monitor whether they are correct in their test responses or when they should adapt some of the responses in the absence of a correct standard? In such cases, students can also deduce predictive cues from analysing their own test‐taking behavior (e.g. analysing how much time or effort it took to generate a response). For multiple‐choice questions, for instance, longer processing times might be interpreted as a higher chance of an incorrect response. In these situations, there is no mental simulation or try‐out test before the judgement, but instead cues arise from actual performance and are used to make inferences about it.

Several medical education studies fall into this category. Eva and Regehr^28^, ^29^ provided students with a series of general knowledge questions while instructing them to only provide an answer when they felt confident about it. Afterwards, students guessed the answers to the questions they initially left blank. The participants’ decision behaviour indicated reasonably accurate self‐monitoring: they mostly deferred answering when their ultimate response was incorrect (only 6.8% of these responses were correct, compared with 72.6% correct on the immediately answered questions). Also, they took longer to decide whether they were confident to answer a question, when they responded to it incorrectly. This shows that students interpreted their longer decision times as a sign of less knowledge. Research by McConnell *et al*.^30^ extended this approach to the high‐stakes Medical Council of Canada Qualifying Examination Part I and analysed (i) the time needed to respond to questions, (ii) the number of questions students identified as needing further consideration (i.e. questions that were ‘flagged’) and (iii) the likelihood of changing their initial response. All three of these indices were related to correctness of responses and indicated proof of accurate self‐monitoring: compared with correct answers, students took longer to provide incorrect answers, were more likely to flag these and were more likely to change their initial answer. High performing examinees also showed greater differentiation on these indices than poor performing examinees. These findings exemplify the value of control cues, such as response time and the decision to change an initial answer, as a basis for monitoring judgements. Teaching students explicitly about the predictive value of behavioural cues such as decision times and how to use them to inform monitoring judgements is a possibly fruitful strategy to improve monitoring and regulation during test taking.

## Application of Cue Diagnosticity and Cue Utilisation in Medical Education

Introduction of the cue utilisation framework to medical education opens new avenues in educational research and practice. We will divide our recommendations into those related to gaining conceptual knowledge (i.e. related to basic or clinical sciences) and those related to clinical reasoning.

### Gaining conceptual knowledge

The transfer of insights from laboratory work on metamemory to medical education appears particularly feasible when it comes to studying factual basic science knowledge. Given that learning of this knowledge constitutes a large part of undergraduate medical programmes, we see opportunities to scale up research to the medical education context. Based on the evidence outlined above, we predict that self‐generative cue prompts (possibly at a delay after learning) will help students activate predictive cues when learning conceptual knowledge (e.g. from medical textbook chapters or scientific articles). Prompts such as having students generate keywords, write a summary or complete a structured diagram about some studied information fall into this category^19^, ^20^, ^21^ and their application to medical education should be explored. Teachers could have students try out these cue prompts, discuss students’ experiences and preferences and determine their effect. Whether students are then able to *use* these predictive cues to inform their judgements is a question for further research. Research into support techniques, such as the idea unit technique, will provide insight into this matter. Given the complexity of many medical concepts and mechanisms, we do expect support techniques will be necessary to ensure use of predictive cues after cue prompting. Training students to first generate and then recognise and use predictive cues in a more controlled setting (such as a classroom environment focusing on text study) could pave the way for improving predictive cue use during self‐directed learning outside of the classroom, during a lecture or when taking a test. These studies should be designed in such a way as to provide insight into the characteristics of predictive (versus non‐predictive) cues in each of these settings.

Note that increased use of cue‐prompt interventions may promote a shift of instruction on reflection towards a focus on specific tasks. Compared with the current situation where the emphasis typically lies on reflection across learning tasks, skills, courses or internships (e.g. through a portfolio)^31^, this new wave will zoom in on the micro‐level of monitoring and regulation of a single learning task. This, of course, does not exclude the possibility of applying the predictive cue prompt approach to the more general level of a course or a curriculum. It does, however, entail a shift towards focusing on monitoring and regulation accuracy.^32^

Finally, researchers should explicitly decide whether they desire to influence absolute or relative monitoring accuracy and should design their predictive cue prompt accordingly, such as by placing emphasis on comparison of cues for relative accuracy (e.g. keyword generation prompts) or emphasis on analysing absolute quality of knowledge for absolute accuracy (e.g. idea unit prompts).

### Clinical reasoning

Contrary to students’ monitoring judgements of processing conceptual knowledge, very little is known about the cues students use when monitoring clinical reasoning. What is known about cue use during clinical reasoning is based on research by medical experts. For example, experts are known to interpret their own slowing down during clinical practice^33^ as a cue for processing difficulty and as a sign to adjust their behaviour (e.g. seek help from a colleague). This slowing down is interpreted as the expert reaching the ‘edges of their automaticity’. However, the slowing down cue depends on expert automaticity and is of limited utility for novices.

De‐biasing strategies have been put forward as a means to improve students’ and trainees’ monitoring of clinical reasoning.^34^ These strategies raise awareness in students and trainees about the biases in their clinical reasoning and urge them to consider alternative reasoning strategies. Although this may be a first step, we pose that it will be insufficient to improve monitoring and regulation of learning. First, de‐biasing is cognitively taxing and may be prohibitively difficult for an early learner for whom clinical decision making is already complex. Second, trainees may lack well‐developed scripts for the alternative strategies. Strategies like ‘consider the opposite’ require adequate knowledge of alternate scripts that most trainees will lack. Lastly, even practising clinicians’ ability to identify biases in reasoning has been questioned.^35^

Instead, we suggest that dedicated research unravelling students’ use of cues during clinical reasoning is the requisite step to start evaluating interventions that aim to improve use of predictive cues (for an example in communication skills training, see Wagner‐Menghin et al^36^). This research could also evaluate the generalisability of concepts of self‐generation and delay that emerged in text‐based educational research. This can be achieved by exploring students’ monitoring judgements through simulated clinical cases (either mental or actual simulation or even reflection on clinical practice) and by explicitly examining the cues that led them to their judgements. Doing so can make students aware of how they monitor their clinical reasoning and decision‐making processes, allowing them to gain insight into what cues they are using and how predictive these cues are.

Because students are probably unaware of some of the cues they use,^8^ merely interviewing students about cue use will not be sufficient to gain a complete (or accurate) picture. Experiments attempting to manipulate the cues students use during clinical reasoning can aid in providing insight into predictive cue use during clinical reasoning. Some studies in clinical reasoning can be seen through this lens. For instance, studies on cardiac diagnosis using a high‐fidelity simulator have identified that trainees rely excessively on the information provided from a short clinical stem. In these studies, participants were unable to overcome either a biased stem^37^ or their own initially misguided impression^38^ when performing a cardiac physical examination. These experiments suggest that trainees were unable to rely on cues from their physical examination and instead overvalued less predictive cues from clinical history in forming their diagnostic impressions. The wide range of reported confidence, with both under‐ and overconfidence, also indicates these trainees are attending to non‐predictive cues when forming judgements of their accuracy in cardiac diagnosis.

After identifying the sources of monitoring judgements during clinical reasoning, the next step in developing predictive cue prompts would be to provide external support to improve the use of cues. This could be implemented by tutors who explore the sources of trainees’ monitoring of their diagnostic impressions during a simulated clinical case or clinical examination. Students and teachers could jointly identify the cues used by students and adjust them to optimise which clinical features are being used to arrive at a diagnosis and how they are being evaluated to generate a monitoring judgement. In clinical reasoning, judgement of performance relative to a minimum standard is of particular importance. Students should learn to monitor whether they can safely treat a patient and have the knowledge and skills to do so. This should be incorporated in development of predictive cue prompts (e.g. by requiring students to reflect on issues such as knowing when to ask for assistance and recognising when their knowledge and skills fail). Once effective cue prompts are defined such instruction could also be implemented through tutor‐independent materials promoting self‐regulated learning.^39^ Feedback on cue use is essential to steer students towards using predictive cues instead of the often more intuitively appealing non‐predictive cues. It would be useful to design studies that allow distinctions to be made between cue diagnosticity and cue utilisation^19^ so as to optimally tailor instruction.

## Conclusion

The cue‐utilisation framework provides important insights into how to improve the accuracy of students’ monitoring judgements so as to help guide learning. Medical students’ and trainees’ accurate judgements of learning and performance critically depend on the availability and use of predictive cues. Cognitive psychology research suggests that self‐generating predictive cues prior to judging learning and performance can improve monitoring and regulation of learning and performance. However, the majority of these studies were based in laboratory‐like learning environments. Medical training provides a unique opportunity for expanding on this research and improving understanding of cue‐utilisation in real‐life settings. A wide range of opportunities exists for research on cues used by medical students and trainees both during self‐directed learning and in their development of clinical reasoning. Research should focus on unravelling current cue use and on designing and testing predictive cue prompts to improve cue use. These cue prompts are likely to incorporate support to help students and trainees evaluate their performance and to become aware of predictive cues and how to use them. This research has the potential to shift educational practice by making explicit how students can accurately judge their learning and clinical decisions. This is likely to have a significant impact not only on acquiring conceptual knowledge but also on clinical learning, because accurate monitoring judgements lead to better diagnoses.

## Contributors

all authors contributed to the concept and structure of the paper. All authors approved the final version of the paper. ADB wrote the introductory section of the paper. ADB and JD wrote the parts related to monitoring and regulation research in cognitive psychology. ADB and RC wrote the sections related to monitoring and regulation in medical education. All authors edited each section of the paper. RC performed the final edit prior to submission.

## Funding

grant support was provided to ADB by the Dutch Science Foundation (NWO VENI grant number 016.115.123).

## Conflicts of interest

there are no competing interests.

## Ethical approval

no human subjects were tested for the development of this manuscript. Therefore, no ethical approval was necessary.
